# Effects of Vaspin on Insulin Resistance in Rats and Underlying Mechanisms

**DOI:** 10.1038/s41598-018-31923-3

**Published:** 2018-09-10

**Authors:** Shiwei Liu, Ruixue Duan, Yaru Wu, Fang Du, Jiaxin Zhang, Xin Li, Shenghui Guo, Meimei Wang, Qi Zhang, Yuanbin Li, Naishi Li

**Affiliations:** 1 Department of Endocrinology, Shanxi DAYI Hospital, Shanxi Medical University, Taiyuan, China; 2 Department of Central Laboratory, Taiyuan Central Hospital, Shanxi Medical University, Taiyuan, China; 3 Department of Graduate School of Shanxi Medical University, Taiyuan, China; 4 Department of Endocrinology, Taiyuan Central Hospital, Shanxi Medical University, Taiyuan, China; 5 Department of Endocrinology, Key Laboratory of Endocrinology, Peking Union Medical College Hospital, Chinese Academy of Medical Science, Beijing, China

## Abstract

Insulin resistance (IR) is the main pathogenesis of metabolic syndrome and a shared pathophysiological change in conditions such as diabetes mellitus, adiposity, hypertension, and atherosclerosis. Visceral adipose tissue-derived serpin (Vaspin) is a newly discovered adipocytokine with insulin-sensitizing and anti-inflammatory effects. To examine if vaspin can improve insulin resistance in rats fed a high-fat diet via the insulin receptor substrate/phosphatidylinositol 3 kinase/protein kinase B/glucose transport (IRS/PI3K/Akt/Glut) and inhibitory κB alpha/nuclear factor-kappa B (IκBα/NF-κB) signalling pathways, thirty male Sprague-Dawley (SD) rats were randomly divided into three groups: the normal control group (NC group, n = 10), high-fat diet group (HFD group, n = 10) and vaspin intervention group (HFD + vaspin group, n = 10). Results showed that intervention with vaspin significantly decreased fasting blood glucose (FBG) and fasting insulin (FINS) concentrations in HFD − fed rats without significantly affecting body weight or triglyceride (TG) or total cholesterol (TC) levels. The areas under the intraperitoneal glucose tolerance test (IPGTT) and the insulin tolerance test (ITT) curves were significantly decreased in HFD + vaspin group compared with the HFD group, and the glucose infusion rate (GIR) showed the same trends. Western blot, real-time polymerase chain reaction (RT-PCR) and immunofluorescence staining showed that vaspin could improve insulin resistance in liver, skeletal muscle and adipose tissue by activating the IRS/PI3K/Akt/Glut signalling pathway and inhibiting the IκBα/NF-κB signalling pathway.

## Introduction

The improvement in people’s living standards and their lifestyle changes, ingesting high calorie food and not getting sufficient exercise, has lead to fat accumulation, obesity and metabolic syndrome, which includes diabetes mellitus, hypertension, hyperlipemia and atherosclerosis^1^. Insulin resistance (IR) is the main pathogenesis of metabolic syndrome and a shared pathophysiological change in diabetes mellitus, adiposity, hypertension and atherosclerosis^2,3^.

Insulin binds to and activates the tyrosine kinase activity of the insulin receptor on the cell surface, leading to the tyrosine phosphorylation and activation of insulin receptor substrate (IRS); activated IRS further activates protein kinase B (Akt) through the activation of phosphatidylinositol 3 kinase (PI3K) and promotes the membrane translocation of glucose transporters (Gluts), thereby reducing blood glucose levels^4–7^. When the insulin signalling pathway is inhibited, insulin no longer produces the desired effects; disruption of IRS tyrosine phosphorylation or an abnormal increase in the phosphorylation of serine/threonine sites blocks insulin signalling and causes cell dysfunction^8,9^. Liver, skeletal muscle and fat are the three main target organs of insulin action^10^.

Studies have shown that inflammatory factors induce IR^11,12^. Nuclear factor-kappa B (NF-κB) is a key regulator of the inflammatory process and plays an important role in the activation of inflammation and pathogenesis^13^. NF-κB is an intracellular target for hyperglycaemia and hyperlipidaemia, and phosphorylation of inhibitory κB alpha (p-IκBα) is the major regulatory step in NF-κB activation^14,15^. Abnormal activation of the NF-κB signalling pathway in liver, skeletal muscle, and adipose tissue affects the insulin signalling pathway, either directly or indirectly leading to IR^16–18^. On the other hand, overexpression of inflammatory molecules downstream of NF-κB, such as tumour necrosis factor alpha (TNF-α) and interleukin 6 (IL-6), can activate the NF-κB pathway, forming a vicious cycle in which the body remains in a chronic state of inflammation that further exacerbates IR^13,19^.

Visceral adipose tissue-derived serpin (vaspin) is an adipocytokine released from Otsuka Long-Evans Tokushima Fatty (OLETF) rat visceral adipose tissue that was identified in 2005 by Hida^20^. Rat, mouse, and human vaspins are made up of 392, 394, and 395 amino acids, respectively; they exhibit approximately 40% homology with α1-antitrypsin and are related to the serine protease inhibitor family. Previous studies found that at 30 weeks of age, OLETF rats exhibit significant IR and a significant increase in serum vaspin levels; this increase was confirmed in humans: the concentration of vaspin was significantly higher in patients with pre-diabetes and diabetes than in those without diabetes^20,21^. Insulin sensitivity in diabetic rats could be improved by vaspin intervention, and blood glucose eventually returned to normal^11^. At the same time, vaspin exerts an anti-inflammatory effect^22,23^. In vascular smooth muscle cells, vaspin inhibits the activation of NF-κB/protein kinase C theta (PKCθ) by reactive oxygen species (ROS) and inhibits the expression of intercellular adhesion molecule-1 (ICAM-1) induced by TNF-α^24^. Vaspin also inhibits NF-κB activation and the expression of adhesion molecules induced by TNF-α and IL-1 to protect vascular endothelial cells^25^.

Studies have showed that vaspin has anti-insulin resistance and anti-inflammatory activity. However, whether vaspin influences the insulin signalling pathway and inflammation-related pathways to improve IR in peripheral tissues is still unknown. Therefore, in this study, we used a high-fat diet (HFD) to establish an animal model of IR and performed hyperinsulinaemic-euglycaemic clamp, western blot, real-time polymerase chain reaction (RT-PCR) and immunofluorescence experiments to explore the ability of vaspin to improve IR through the IRS/PI3K/Akt/Glut and IκBα/NF-κB signalling pathways.

## Results

### Effects of vaspin on body weight and other related indexes in SD rats

**Figure 1:**
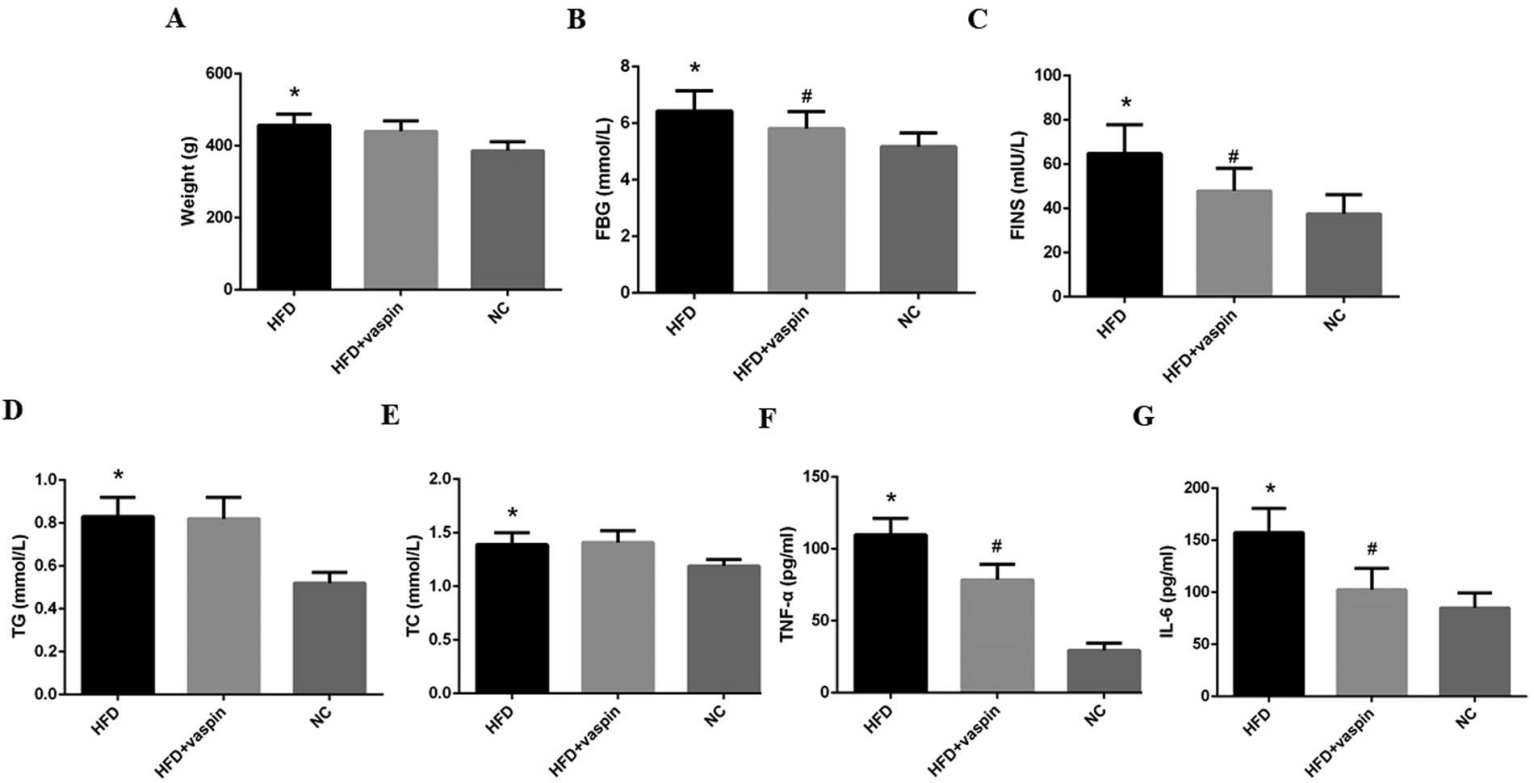
Effects of vaspin on rat body weight and other metabolic indexes, n = 10/group. At the end of the vaspin intervention, rat body weight and other metabolic indexes were detected. (**A**) Body weight. (**B**) Fasting blood glucose. (**C**) Fasting insulin. (**D**) Triglyceride. (**E**) Total cholesterol. (**F**) Tumour necrosis factor alpha. (**G**) Interleukin 6. Data are presented as the mean ± SD of at least three independent experiments. One-way analysis of variance, followed by the least significant difference (LSD) t-test and Tukey’s test, was used to calculate differences among the various study groups. *P < 0.05 compared with the NC group; ^#^*P* < 0.05 compared with the HFD group.

After vaspin intervention, rats were weighed, and the levels of serum metabolic indexes were measured. Compared with rats in the normal control group (NC), those in the HFD group showed an increase in body weight, and the serum concentrations of fasting blood glucose (FBG), fasting insulin (FINS), triglyceride (TG) and total cholesterol (TC) were also increased significantly (Fig. 1A–E). However, intervention with vaspin significantly lowered serum FBG and FINS concentrations in HFD − fed rats but had no significant effect on body weight or TG or TC levels (Fig. 1A–E). In addition, vaspin reversed the HFD-induced increases in serum TNF-α and IL-6 levels (Fig. 1F–G). These results suggest that vaspin improves IR, lowers blood glucose, and reduces inflammation.

### Effects of vaspin on the intraperitoneal glucose tolerance test (IPGTT) and insulin tolerance test (ITT)in rats

**Figure 2:**
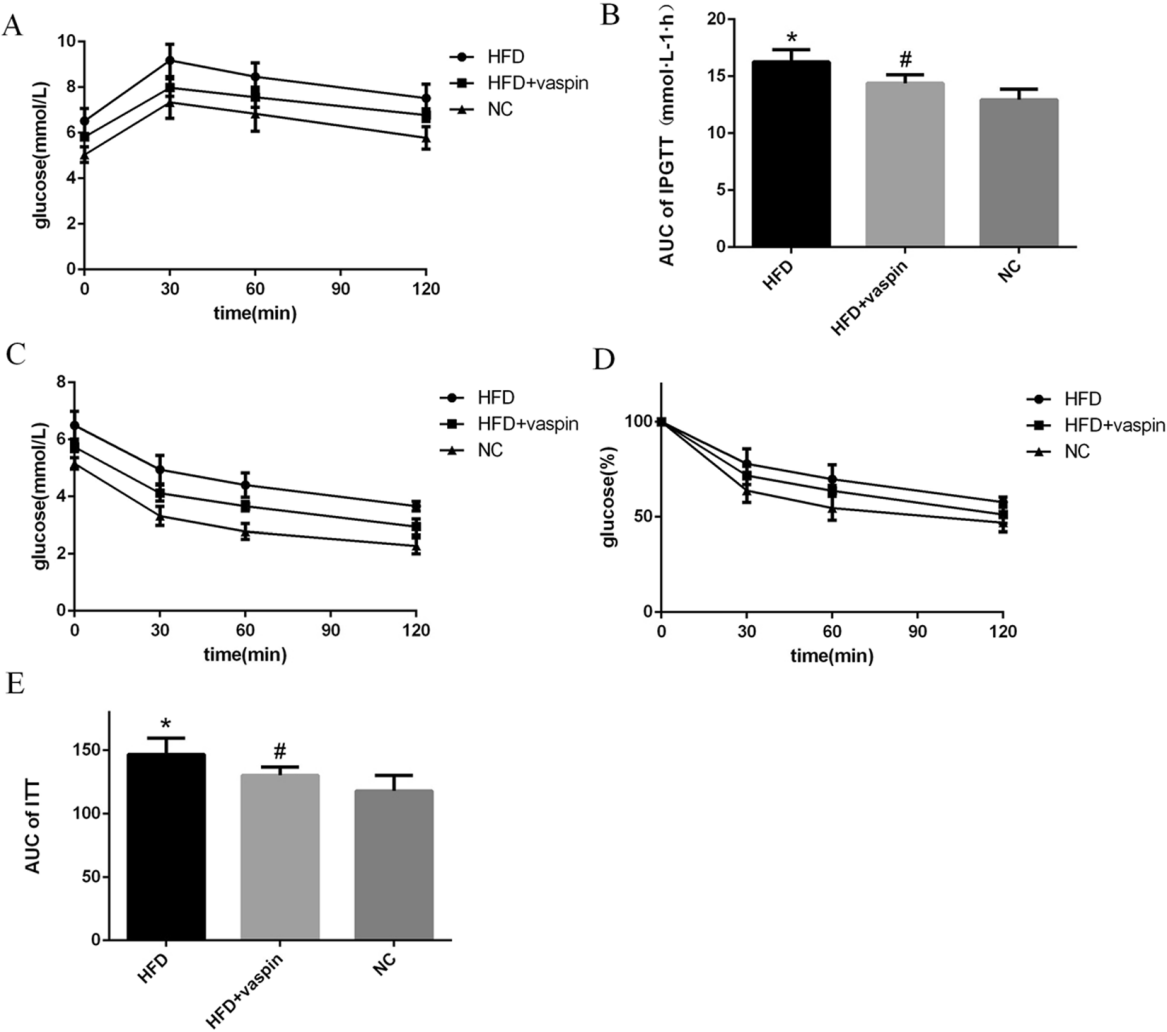
Vaspin improved the glucose and insulin tolerance of HFD − fed rats, n = 10/group. At the end of the vaspin intervention, the IPGTT and ITT were performed in overnight-fasted rats. (**A**) IPGTT. (**B**) Area under the curve of IPGTT. (**C**) ITT. (**D**) The ITT results are shown as a percentage. (**E**) Area under the curve of ITT. Data are presented as the mean ± SD. The results were analysed with one-way analysis of variance, followed by the LSD test and Tukey’s test. *P < 0.05 compared with the NC group; ^#^*P* < 0.05 compared with the HFD group.

The IPGTT was used to assess glucose tolerance in rats after vaspin intervention. The area under the IPGTT curve was increased significantly in the HFD group compared with the NC group but was decreased significantly in the HFD + vaspin group compared with the HFD group (Fig. 2A,B). The results showed that vaspin could improve glucose tolerance in HFD − fed rats. Meanwhile, we also used the ITT to evaluate insulin tolerance after vaspin treatment. Vaspin also improved insulin tolerance in HFD − fed rats, as shown by significant decreases in plasma glucose levels and the area under the ITT curve in the HFD + vaspin group compared with the HFD group (Fig. 2C–E).

### Effects of vaspin on insulin resistance in rats

**Figure 3:**
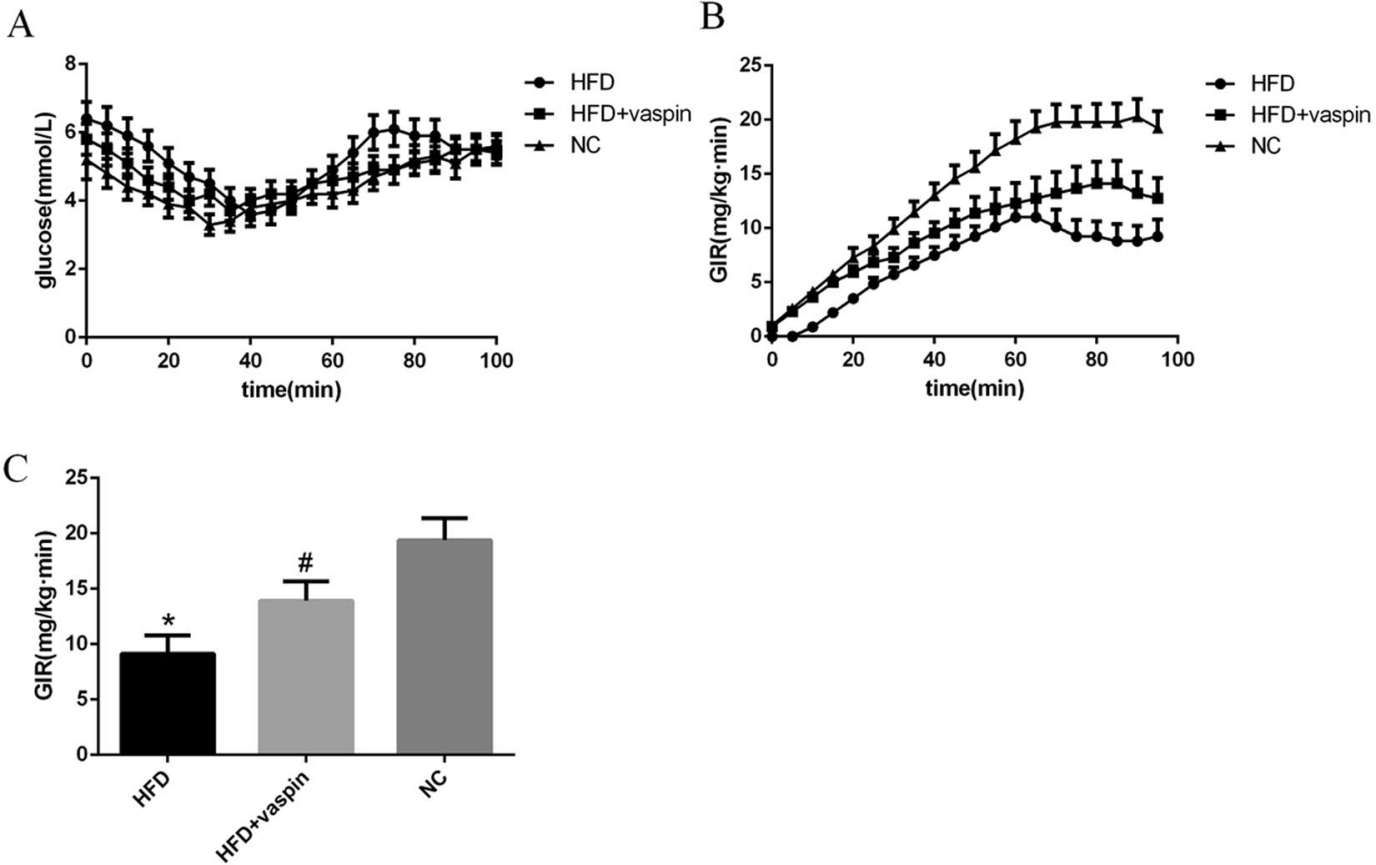
Vaspin improved the insulin resistance of HFD − fed rats, n = 10/group. At the end of the vaspin intervention, the hyperinsulinaemic-euglycaemic clamp test was performed in overnight-fasted rats. (**A**) Plasma glucose level. (**B**) Glucose infusion rate during clamp. (**C**) Glucose infusion rate at steady-state. Data are presented as the mean ± SD. The results were analysed with one-way analysis of variance, followed by the LSD test and Tukey’s test. *P < 0.05 compared with NC group; ^#^*P* < 0.05 compared with HFD group.

The hyperinsulinaemic-euglycaemic clamp test was used to evaluate IR in the peripheral tissues of rats after vaspin intervention, and changes in the insulin sensitivity of rat peripheral tissues were determined according to the glucose infusion rate (GIR) at equilibrium. The results showed that the steady-state GIR decreased significantly in the HFD group compared with the NC group and increased steadily in the HFD + vaspin group compared with the HFD group (Fig. 3A–C). In conclusion, vaspin could improve the insulin sensitivity of peripheral tissues in HFD − fed rats.

### Effects of vaspin on the IRS/PI3K/Akt/Glut signalling pathway in the rat liver

**Figure 4:**
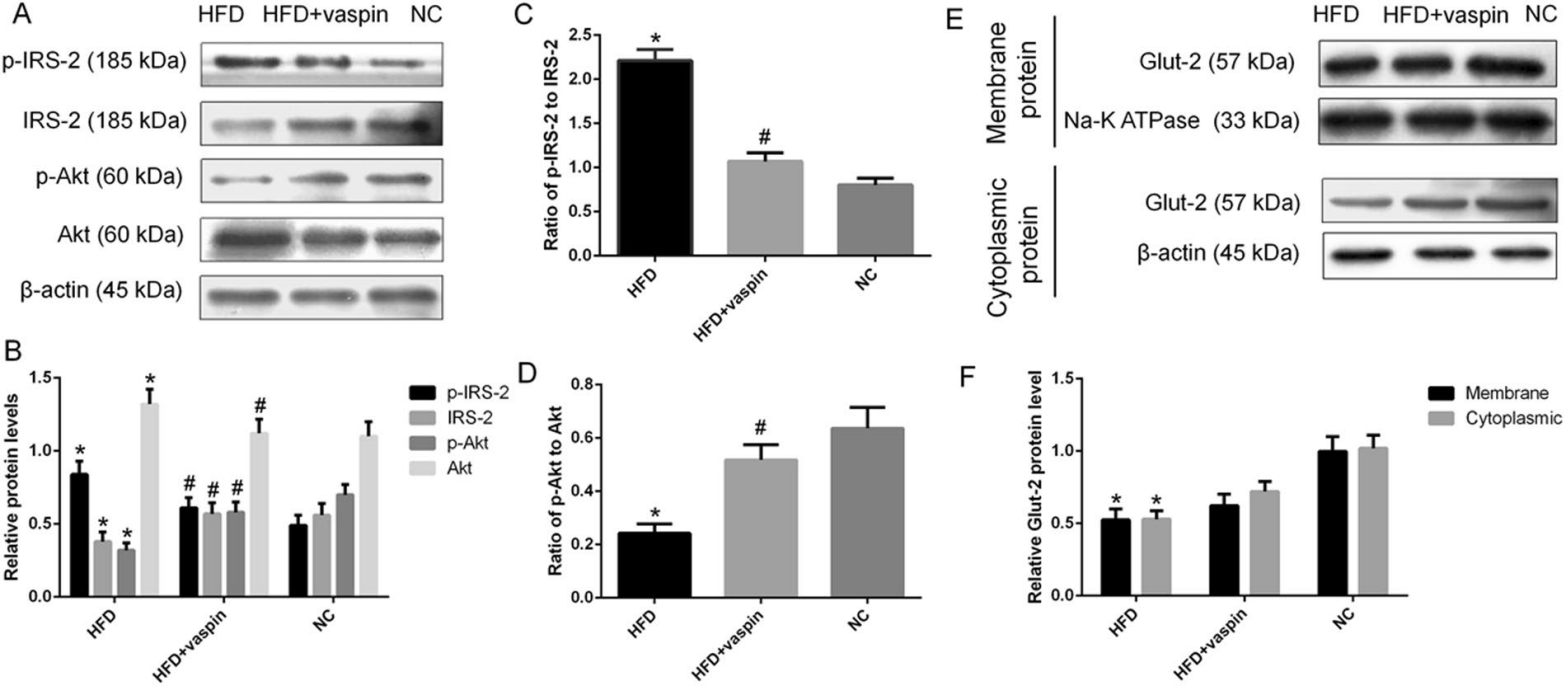
Vaspin promoted IRS/PI3K/Akt/Glut signalling in the liver of HFD − fed rats. Western blot was performed to detect the protein levels of IRS/PI3K/Akt/Glut signalling pathway components in liver tissue. (**A**) Western blot results of p(Ser)-IRS-2, IRS-2, p-Akt and Akt. (**B**) p(Ser)-IRS-2, IRS-2, p-Akt and Akt levels were quantified. (**C**) Ratio of p-IRS-2 to IRS-2. (**D**) Ratio of p-Akt to Akt. (**E**) Western blot results of membrane Glut 2 and cytoplasmic Glut 2. (**F**) Membrane and cytoplasmic Glut 2 levels were quantified. Data are presented as the mean ± SD of at least three independent experiments. The results were analysed with one-way analysis of variance, followed by the LSD test and Tukey’s test. The Kruskal-Wallis test was used to analyse the differences in Glut 2 levels among groups because while the data obeyed a normal distribution, the variance was not uniform. *P < 0.05 compared with the NC group; ^#^*P* < 0.05 compared with the HFD group.

The expression levels of IRS/PI3K/Akt/Glut signalling pathway components in the rat liver were determined by western blot. Compared with the NC group, the HFD group showed a significant increase in the serine phosphorylation of IRS-2 (p-IRS-2) and Akt protein expression and a decrease in IRS-2 expression and the phosphorylation of Akt; these effects could be reversed by vaspin (Fig. 4A,B). Vaspin also decreased the ratio of p-IRS-2 to IRS-2 and increased the ratio of p-Akt to Akt (Fig. 4C,D). At the same time, membrane-localized and cytoplasmic Glut 2 protein levels were downregulated by HFD, and there were small but not significant difference between the HFD and HFD + vaspin groups (Fig. 4E,F). In conclusion, vaspin may improve the insulin sensitivity of the liver in HFD − fed rats by promoting the expression of IRS/PI3K/Akt/Glut signalling pathway components.

### Effects of vaspin on the IRS/PI3K/Akt/Glut signalling pathway in rat skeletal muscle

**Figure 5:**
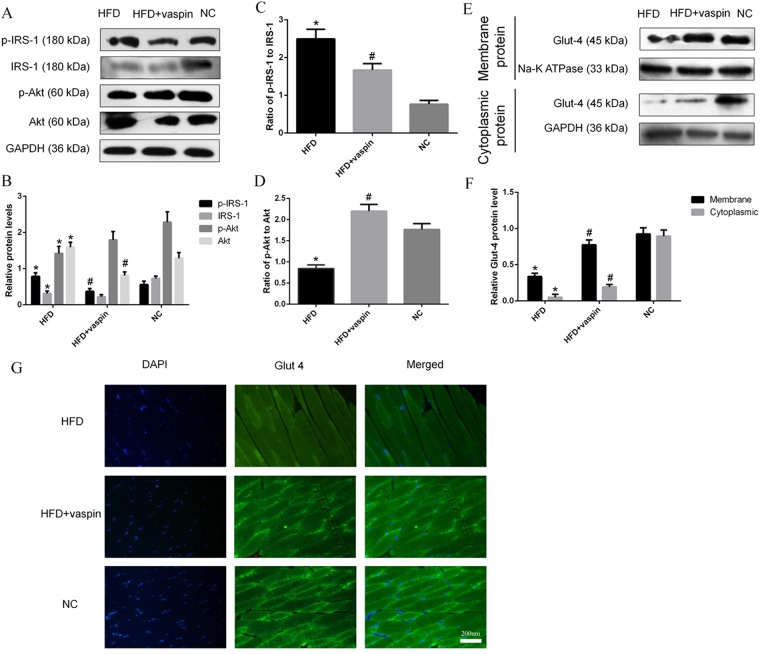
Vaspin promoted IRS/PI3K/Akt/Glut signalling in skeletal muscle of HFD − fed rats. Western blot was performed to detect the protein levels of IRS/PI3K/Akt/Glut signalling pathway components in skeletal muscle. (**A**) Western blot results of p(Ser)-IRS-1, IRS-1, p-Akt and Akt. (**B**) p(Ser)-IRS-1, IRS-1, p-Akt and Akt levels were quantified. (**C**) Ratio of p-IRS-1 to IRS-1. (**D**) Ratio of p-Akt to Akt. (**E**) Western blot results of membrane and cytoplasmic Glut 4. (**F**) Membrane and cytoplasmic Glut 4 levels were quantified. (**G**) Immunofluorescence staining of Glut 4. Data are presented as the mean ± SD of at least three independent experiments. Differences among groups were compared with one-way analysis of variance, followed by the LSD test and Tukey’s test. *P < 0.05 compared with the NC group; ^#^*P* < 0.05 compared with the HFD group.

The expression levels of IRS/PI3K/Akt/Glut signalling pathway components in skeletal muscle were detected by western blot and immunofluorescence staining. The results showed that vaspin significantly decreased the serine phosphorylation of IRS-1 and the ratio of p-IRS-1 to IRS-1 induced by HFD and increased the ratio of p-Akt to Akt (Fig. 5A–D). Meanwhile, both membrane-localized and cytoplasmic Glut 4 protein levels were downregulated by HFD and upregulated after vaspin intervention (Fig. 5E,F). Immunofluorescence assays yielded the same results: Glut 4 fluorescence was significantly attenuated in the HFD group and significantly enhanced in the HFD + vaspin group (Fig. 5G). These results indicate that vaspin may improve the insulin sensitivity of skeletal muscle in HFD − fed rats by promoting the expression of IRS/PI3K/Akt/Glut signalling molecules.

### Effects of vaspin on the IRS/PI3K/Akt/Glut signalling pathway in rat adipose tissue

**Figure 6:**
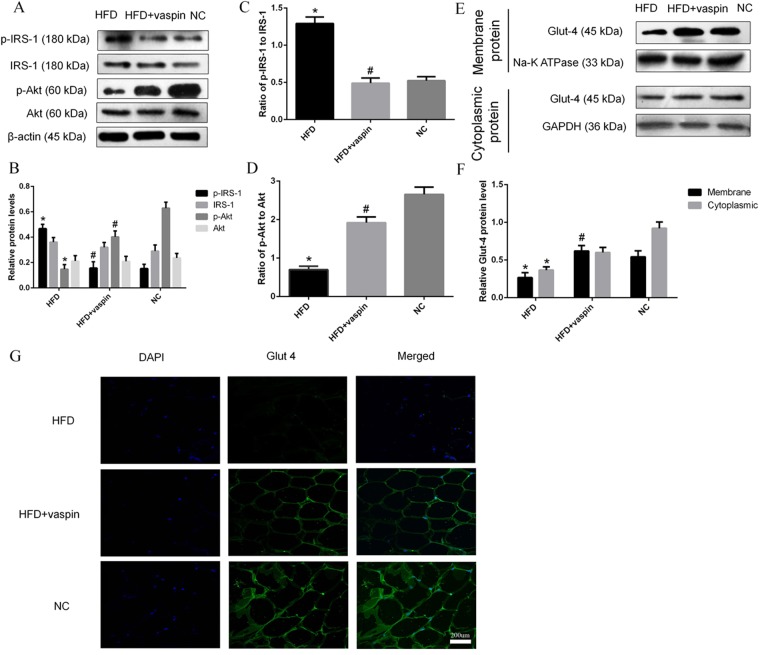
Vaspin promoted IRS/PI3K/Akt/Glut signalling in adipose tissue of HFD − fed rats. Western blot was performed to detect the protein levels of IRS/PI3K/Akt/Glut signalling pathway components in adipose tissue. (**A**) Western blot results of p(Ser)-IRS-1, IRS-1, p-Akt and Akt. (**B**) p(Ser)-IRS-1, IRS-1, p-Akt and Akt levels were quantified. (**C**) Ratio of p-IRS-1 to IRS-1. (**D**) Ratio of p-Akt to Akt. (E) Western blot results of membrane and cytoplasmic Glut 4. (**F**) Membrane and cytoplasmic Glut 4 levels were quantified. (**G**) Immunofluorescence staining of Glut 4. Data are presented as the mean ± SD of at least three independent experiments. One-way analysis of variance, followed by the LSD test and Tukey’s test, was used to compare differences among groups. The cytoplasmic Glut 4 data obeyed a normal distribution, but the variance was not uniform; thus, the Kruskal-Wallis test was used to analyse the differences. *P < 0.05 compared with the NC group; ^#^*P* < 0.05 compared with the HFD group.

The expression levels of IRS/PI3K/Akt/Glut signalling pathway components in adipose tissue were detected by western blot and immunofluorescence staining. Compared with the NC group, the HFD group showed a significant increase in the serine phosphorylation of IRS-1 and the ratio of p-IRS-1 to IRS-1 and an obvious decrease in p-Akt levels and the ratio of p-Akt to Akt. However, vaspin inhibited these effects induced by HFD (Fig. 6A–D). Levels of membrane-localized Glut 4 decreased significantly after HFD and increased upon vaspin intervention. Cytoplasmic Glut 4 protein levels were higher in the HFD + vaspin group than in the HFD group, but this difference was not statistically significant (Fig. 6E,F). Immunofluorescence staining showed that Glut 4 fluorescence was significantly attenuated in the HFD group and significantly enhanced in the HFD + vaspin group (Fig. 6G). All these results indicate that vaspin may improve the insulin sensitivity of fat in HFD − fed rats by promoting the expression of IRS/PI3K/Akt/Glut signalling molecules.

### Effects of vaspin on the IκBα/NF-κB signalling pathway in the rat liver

**Figure 7:**
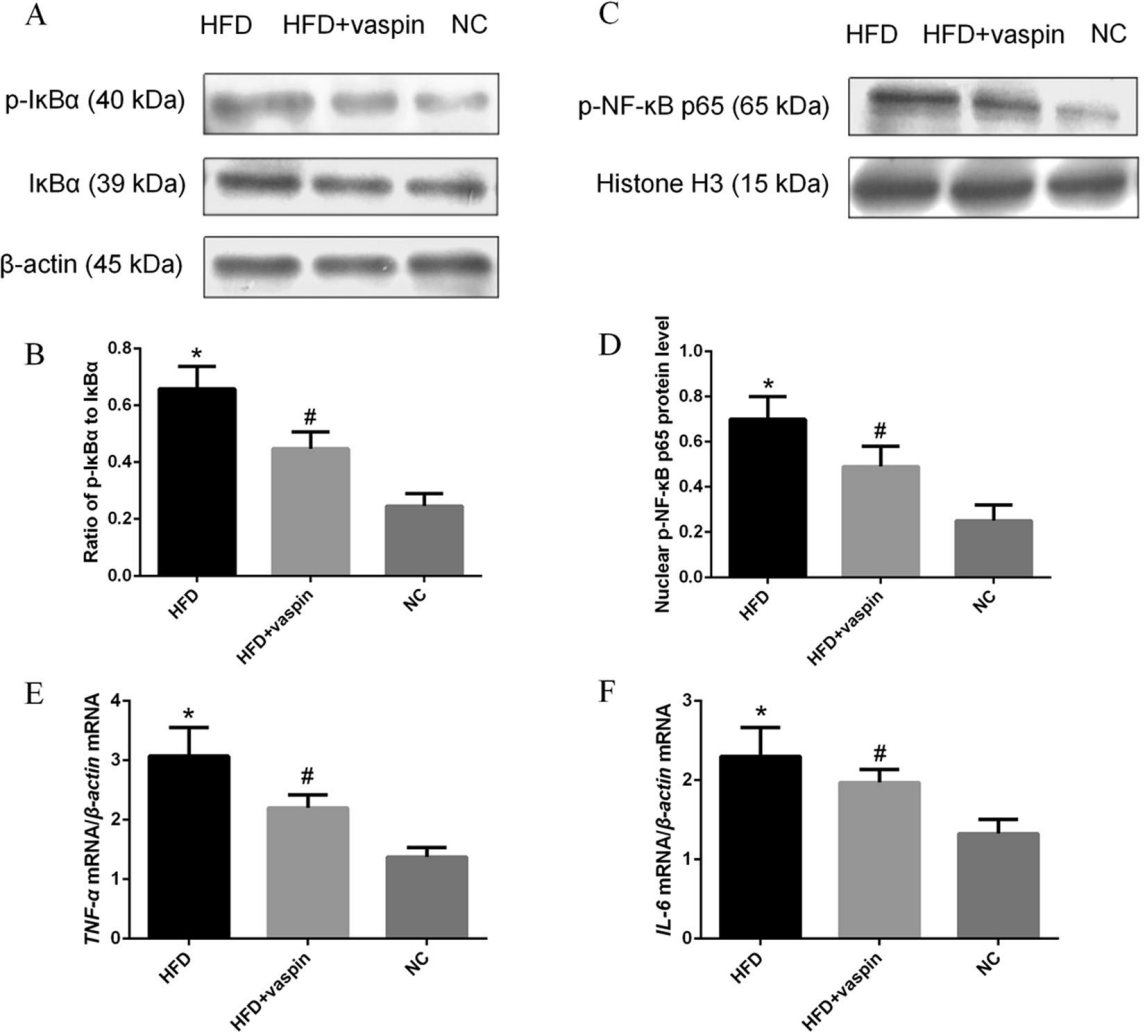
Vaspin inhibited the IκBα/NF-κB signalling pathway in the liver of HFD − fed rats. Western blot and RT-PCR were used to detect the levels of IκBα/NF-κB signalling pathway components in liver tissue. (**A**) Western blot results of p-IκBα and IκBα. (**B**) Ratio of p-IκBα to IκBα. (**C**) Western blot results of p-NF-κB p65. (**D**) Quantification of p-NF-κB p65 protein level. (**E**) Relative *TNF-α* mRNA level. (**F**) Relative *IL-6* mRNA level. Data are presented as the mean ± SD of at least three independent experiments. The results were analysed with one-way analysis of variance, followed by the LSD test and Tukey’s test. *P < 0.05 compared with the NC group; ^#^*P* < 0.05 compared with the HFD group.

The expression levels of NF-κB signalling pathway components and downstream inflammatory molecules in rat liver were determined by western blot and RT-PCR. The ratio of p-IκBα to IκBα in the liver was higher in the HFD group than in the NC group, and vaspin could reverse this effect (Fig. 7A,B). The results showed that the phosphorylation of NF-κB p65 (p-NF-κB p65) in the nucleus was increased in the HFD group and decreased after vaspin intervention (Fig. 7C,D). At the same time, the mRNA levels of the downstream inflammatory factors *TNF-α* and *IL-6* were increased in HFD − fed rats and were decreased after vaspin intervention (Fig. 7E,F). Therefore, vaspin can inhibit the expression of IκBα/NF-κB signalling pathway components and reduce inflammation in the liver of HFD − fed rats.

### Effects of vaspin on the IκBα/NF-κB signalling pathway in rat skeletal muscle

**Figure 8:**
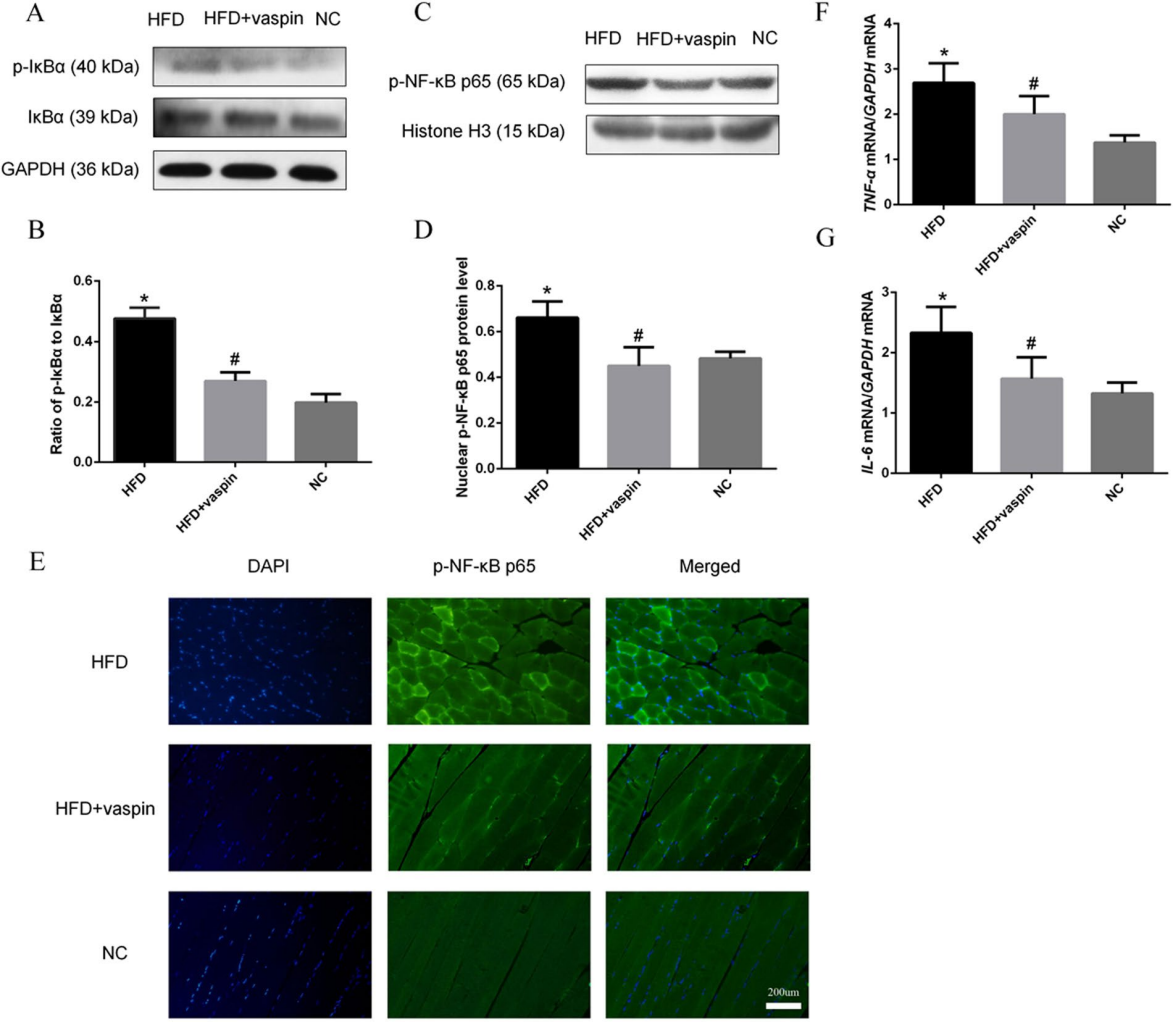
Vaspin inhibited the IκBα/NF-κB signalling pathway in skeletal muscle of HFD − fed rats. Western blot, RT-PCR and immunofluorescence staining were used to detect the levels of IκBα/NF-κB signalling pathway components in skeletal muscle. (**A**) Western blot results of p-IκBα and IκBα. (**B**) Ratio of p-IκBα to IκBα. (**C**) Western blot results of p-NF-κB p65 protein level. (**D**) Quantification of p-NF-κB p65 protein levels. (**E**) Immunofluorescence staining of p-NF-κB p65. (**F**) Relative *TNF-α* mRNA level. (**G**) Relative *IL-6* mRNA level. Data are presented as the mean ± SD of at least three independent experiments. The results were analysed with one-way analysis of variance, followed by the LSD test and Tukey’s test. *P < 0.05 compared with the NC group; ^#^*P* < 0.05 compared with the HFD group.

The expression levels of NF-κB signalling pathway components and downstream inflammatory molecules in rat skeletal muscle were determined by western blot, RT-PCR and immunofluorescence staining. The results showed that the ratio of p-IκBα to IκBα in skeletal muscle was higher in the HFD group than in the NC group, and this increase was reversed by vaspin (Fig. 8A,B). Nuclear p-NF-κB p65 protein levels in skeletal muscle cells were higher in the HFD group than in the NC group, and vaspin intervention significantly reversed this effect (Fig. 8C,D). Immunofluorescence analysis also showed that p-NF-κB p65 protein was upregulated significantly by HFD, and vaspin inhibited this effect (Fig. 8E). Meanwhile, vaspin downregulated the mRNA levels of downstream inflammatory factors such as *TNF-α* and *IL-6* induced by HFD (Fig. 8F,G). All these data indicate that vaspin can inhibit the expression of IκBα/NF-κB signalling pathway components and reduce inflammation in rat skeletal muscle.

### Effects of vaspin on the IκBα/NF-κB signalling pathway in rat adipose tissue

**Figure 9:**
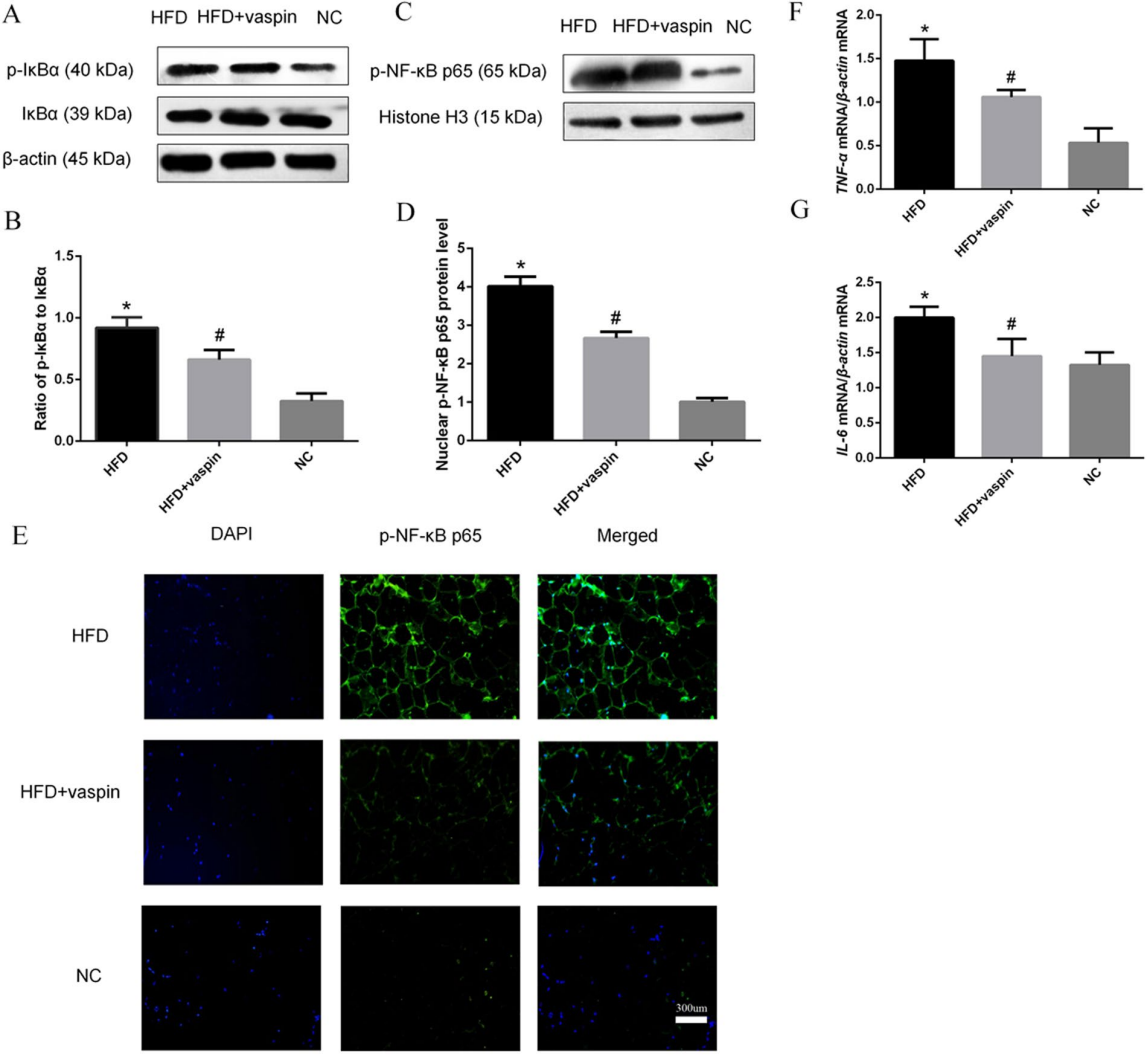
Vaspin inhibited the IκBα/NF-κB signalling pathway in adipose tissue of HFD − fed rats. (**A**) Western blot results of p-IκBα and IκBα. (**B**) Ratio of p-IκBα to IκBα. (**C**) Western blot results of p-NF-κB p65. (**D**) Quantification of p-NF-κB p65 protein level. (**E**) Immunofluorescence staining of p-NF-κB p65. (**F**) Relative *TNF-α* mRNA level. (**G**) Relative *IL-6* mRNA level. Data are presented as the mean ± SD of at least three independent experiments. The results were analysed with one-way analysis of variance, followed by the LSD test and Tukey’s test. *P < 0.05 compared with the NC group; ^#^*P* < 0.05 compared with the HFD group.

The expression levels of NF-κB signalling pathway components and downstream inflammatory molecules in adipose tissue were determined by western blot, RT-PCR and immunofluorescence staining. Compared with the NC group, the HFD group showed a significant increase in the ratio of p-IκBα to IκBα in adipose tissue, and vaspin inhibited this effect (Fig. 9A,B). Both the western blot and immunofluorescence analyses showed that nuclear p-NF-κB p65 levels in adipocytes were increased in the HFD group and decreased after vaspin intervention (Fig. 9C–E). Meanwhile, the mRNA levels of *TNF-α* and *IL-6* increased in fat tissue in HFD − fed rats and decreased significantly in those treated with HFD + vaspin (Fig. 9F,G). Together, these observations demonstrate that vaspin inhibits the expression of IκBα/NF-κB signalling pathway components and reduces inflammation in rat adipose tissue.

## Discussion

IR is a central feature of metabolic syndrome that significantly increases the risk of cardiovascular diseases such as atherosclerosis^26^. Vaspin is an adipocytokine that was isolated from visceral white adipose tissue of OLETF rats in 2005 and belongs to the serine protease inhibitor family^20^. Vaspin levels increased significantly when the body weight and IR of OLETF rats peaked, and vaspin secretion began to decrease as diabetes progressed^20^. These data suggest that the increase in vaspin may be a compensatory response to obesity and IR in order to reduce the associated metabolic stress. We hypothesized that vaspin can regulate insulin sensitivity in the body through certain metabolic links. Therefore, a HFD − fed animal model was used to investigate the effects of vaspin on IR and related mechanisms.

A number of independent studies have indicated that vaspin is significantly increased in the serum of obese individuals, and vaspin levels were significantly related to body mass index (BMI) and waist-to-hip ratio (WHR)^27,28^. Klöting *et al*.^29^ also found that the central administration of vaspin to rats subdues appetite, thereby reducing body weight. In this study, a downward trend in body weight could be seen in HFD − fed rats treated with vaspin, but the differences were not statistically significant; we considered that this finding could be related to the route of vaspin administration. Vaspin was injected intraperitoneally, and the level of vaspin that reached the central nervous system via circulation was too low to inhibit feeding behaviour. This finding may also be related to the number of rats in the experiment, and this difference may become statistically significant as the sample size increases.

Studies have shown that vaspin can improve glucose tolerance and insulin sensitivity in rats with diet-induced obesity and plays an important role in lowering blood glucose and reducing the insulin concentration^20,30^. The present study found that vaspin intervention could significantly reduce the serum levels of FBG and FINS in rats with HFD-induced IR. Studies have also shown that serum vaspin levels are significantly associated with TG and high-density lipoprotein cholesterol (HDL-C) in type 2 diabetes mellitus (T2DM)^31,32^. Choi *et al*.^33^ also found that vaspin levels increased with increasing TG levels. Therefore, this study also investigated the effects of vaspin on lipid metabolism, and the results showed that vaspin had no significant effect on serum TG or TC levels in HFD − fed rats. Weixia Jian *et al*.^34^ found that vaspin levels did not correlate with TG or TC levels in either control individuals or those with T2DM, and Sperling M *et al*.^35^ also showed that vaspin levels did not correlate with TG or TC levels in obese individuals. Therefore, vaspin may have less of an effect on lipid metabolism, as indicated by the current results on the effects of vaspin on TG and TC in rats with IR.

Vaspin is regarded as a potential adipocytokine with insulin-sensitizing effects. Heiker *et al*.^36^ reported that vaspin improved glucose tolerance of mice but could not improve insulin sensitivity. Others, however, confirmed that vaspin could significantly improve glucose tolerance and insulin sensitivity in mice^20,30,37^ In this study, the IPGTT and ITT results showed that vaspin could significantly improve glucose tolerance and insulin tolerance in insulin-resistant rats. The hyperinsulinaemic-euglycaemic clamp technique, the gold standard for evaluating the insulin sensitivity of peripheral tissues *in vivo*, was also used. With this technique, the negative feedback between glucose and insulin *in vivo* can be broken by the simultaneous injection of controlled concentrations of exogenous insulin and glucose at specific rates; thus, plasma exogenous insulin levels were maintained at a high level, while blood glucose remained at the basal steady-state level, and the exogenous GIR under this condition is equivalent to the utilization of glucose in peripheral tissues^38^. The results showed that the GIR in the HFD group was significantly lower than that in the NC group, but it increased after vaspin intervention. These data proved that vaspin can improve IR in the peripheral tissues of HFD − fed rats.

Hida *et al*.^20^ showed that vaspin could improve the insulin sensitivity of white adipose tissue by regulating the expression of genes related to metabolic disorders such as Glut, leptin, resistin and adiponectin. Heiker *et al*.^36^ suggested that vaspin improves glucose tolerance solely through its serine protease inhibitory activity. Therefore, according to the experimental results, we speculated that vaspin may regulate the proteolysis cascade and the gene expression levels of metabolic pathway components in insulin target tissues, hence affecting glucose uptake capacity and insulin sensitivity and finally achieving the beneficial effects on glucose metabolism. The results of our studies showed that vaspin could reduce the ratio of p-IRS-1 (p-IRS-2) to IRS-1 (IRS-2) in liver, skeletal muscle and adipose tissue but increase the ratio of p-Akt to Akt and the membrane translocation of Glut-4 in skeletal muscle and adipose tissue. However, vaspin had little effect on the membrane translocation of Glut-2 in liver tissue; we speculated that the vaspin intervention time was not sufficiently long to cause obvious changes. The improvements induced by vaspin may be more obvious after longer interventions. Nonetheless, the study demonstrates that vaspin can improve glucose tolerance and insulin sensitivity by promoting the IRS/PI3K/Akt/Glut signal transduction pathway in the peripheral tissues of HFD − fed rats.

In addition to its insulin-sensitizing effect, vaspin also has an anti-inflammatory effect^20^. Vaspin can exert its anti-inflammatory effect by inhibiting the expression of the proinflammatory cytokines leptin, resistin, and TNF-α in mouse white adipose tissue^20^. Vaspin can reduce the inflammatory response of vascular smooth muscle cells induced by TNF-α by inhibiting ROS/PKC/NF-κB signal transduction^24^. Our previous study found that vaspin can inhibit NF-κB activation and the expression of downstream inflammatory molecules mediated by the proinflammatory cytokines TNF-α and IL-1, thereby inhibiting inflammation in vascular endothelial cells^25^. This study found that vaspin could act on the IκBα/NF-κB signalling pathway in the liver, skeletal muscle and adipose tissue, inhibiting the normal nuclear translocation of NF-κB and reducing the serum expression of the inflammatory factors TNF-α and IL-6, to dampen the inflammatory state. Vaspin may also rely on its own serine protease inhibitor activity to prevent the production of proinflammatory factors and thus exert anti-inflammatory effects^23^.

NF-κB, a key regulator of inflammation, plays an important role in inflammatory activation and in the inflammatory signalling cascade^39^. Yuan and others^40^ found that a high dose of salicylic acid can inhibit the activity of IκB kinase beta (IKK-β) and reverse hyperglycaemia and IR in obese mice. Hotamisligil *et al*.^41^ also found that salicylate helps improve glucose intolerance in patients. All of these studies demonstrate the beneficial effects of inhibiting the NF-κB signalling pathway on improving insulin sensitivity *in vivo*. Activation of the intracellular NF-κB signalling pathway can promote the activation of downstream proinflammatory factors such as TNF-α and IL-6, which decrease transduction of the insulin signalling pathway and inhibit insulin sensitivity^42^. Therefore, we believe that the ability of vaspin to inhibit the expression of NF-κB signalling pathway components and downstream inflammatory factors in the liver, skeletal muscle and adipose tissue can not only reduce the *in vivo* inflammatory state but also play an important role in improving insulin sensitivity. Studies have confirmed that NF-κB can be activated by the classical IKK/IκBα and PI3K/Akt signalling pathways in endothelial cells^43,44^. However, the interaction between the PI3K/Akt and NF-κB signalling pathways in peripheral tissues remains to be further studied.

In conclusion, vaspin is an adipocytokine derived from visceral adipose tissue that is closely related to obesity and metabolic syndrome; vaspin can improve the insulin resistance of rats by activating the IRS/PI3K/Akt/Glut signalling pathway and inhibiting the IκBα/NF-κB inflammatory pathway. Therefore, further research on vaspin will bring new hope for the treatment of T2DM and metabolic syndrome.

## Materials and Methods

### Animals

Thirty 8 weeks old (180–200 g) male Sprague-Dawley (SD) rats were purchased from Beijing Vital River Laboratory Animal Technology Co., Ltd. (Beijing, China). The rats were housed in the Laboratory Animal Center of Shanxi Medical University; all SPF-grade animals were housed and fed in a barrier environment. All animals were housed in wire-bottomed cages under a 12 h/12 h light/dark cycle starting at 07:00 AM at a controlled temperature (22–26 °C) and a relative humidity of 40–60%; the animals had free access to standard lab chow and tap water. The use of experimental animals was in strict accordance with the Regulations for the Administration of Affairs Concerning Experimental Animals, and the study protocol was approved by the Institutional Animal Care and Use Committee of Shanxi Medical University.

### Induction of insulin resistance and treatment with vaspin

After one week of adaptive feeding, the rats were randomly assigned to a normal diet group (NC group, n = 10) and a high-fat diet group (HFD group, n = 20). The normal diet contained 57% carbohydrate, 18% protein, and 25% fat; the HFD contained 37% carbohydrate, 13% protein and 50% fat. After 16 weeks of dietary manipulation, the rats in the HFD group were randomly divided into the HFD group (n = 10) and the HFD + vaspin group (n = 10). Rats in the HFD + vaspin group were treated with 320 ng/ml (3 ml/kg) vaspin intraperitoneally once daily for 8 weeks. Rats in the NC and HFD groups were given the same volume of normal saline solution (as vehicle control) for the same duration as vaspin.

### Detection of blood biochemical indexes

At the end of the vaspin intervention, fasting blood samples were collected from overnight-fasted rats. Immediately after complete anaesthesia by aether, approximately 1.2 ml of blood was collected from the fundus venous plexus into a centrifuge tube. Blood was centrifuged (4500 rpm/min; 4 °C; 15 min), and the plasma was stored in aliquots at −80 °C for further analyses. Blood glucose was measured via the tail vein using a Freestyle Blood Glucose Meter (Johnson & Johnson, New Jersey, USA). Fasting plasma insulin (FINS), total cholesterol (TC) and triglyceride (TG) were determined using an automated analyser from Shenzhen Icubio Biomedical Technology Co., Ltd. (Shenzhen, China). TNF-α and interleukin-6 (IL-6) secretion was measured with a commercial enzyme-linked immunosorbent assay (ELISA) kit from R&D Systems (Minneapolis, MN) according to the manufacturer’s instructions.

### Intraperitoneal glucose tolerance test (IPGTT)

The intraperitoneal glucose tolerance test (IPGTT) was performed in overnight-fasted rats at the end of the intervention. Each experimental animal received a single dose of 1.5 g/kg body weight of 50% glucose solution (Wuhan Fuxing Biological Pharmaceutical Co., Ltd, Wuhan, China) via intraperitoneal injection. Blood samples were obtained from the tail vein, and glucose values were measured by a glucose metre before glucose loading (t = 0) and at 30, 60, and 120 min after glucose administration. The area under the curve for glucose (AUC glucose) was used to assess the glucose tolerance of rats in different groups.

### Insulin tolerance test (ITT)

At the end of the intervention, overnight-fasted rats were intraperitoneally injected with Recombinant Human Insulin (1 IU/kg body weight; Humulin R, Eli Lilly and Company, Indianapolis, IN, USA). Blood samples were obtained from the tail vein. The glucose values were measured with the glucose metre before loading (t = 0) and at 30, 60, and 120 min after insulin administration.

### Hyperinsulinaemic-euglycaemic clamp test

The hyperinsulinaemic-euglycaemic clamp test is the gold standard method to assess insulin sensitivity. Rats were fasted overnight and anaesthetized with 50 mg/kg body weight sodium pentobarbital (Beijing Solarbio Cable Technology Co. Ltd, Beijing, China) via intraperitoneal injection, with subsequent maintenance doses of approximately 5–10 mg/kg provided as needed at 20 to 30 min intervals. A three-way pipe was placed in the right jugular vein for the infusion of insulin and 20% glucose. The hyperinsulinaemic-euglycaemic clamp test was conducted in rats with a primed three-way pipe under continuous infusion of human insulin (priming followed by infusion at 10 mIU/kg·min). Blood glucose was measured at time 0, immediately after the initial injection of insulin, and at 5–10 min intervals. When blood glucose dropped below 5–6 mmol/L, the peristaltic pump was started, and 20% glucose was infused at a variable rate to maintain the concentration at (5.5 ± 0.5) mmol/L for 5 consecutive time points. Changes in the insulin sensitivity of peripheral tissues were assessed according to the glucose infusion rate (GIR): GIR (mg/kg·min) = rate (μl/min) × glucose concentration (g/ml) ÷ 1000 ÷ B.W. (g). The insulin level was used to evaluate the bidirectional regulatory effects of islet β cells. Liver, hind leg skeletal muscle and epididymal fat were excised and snap-frozen in liquid nitrogen. Tissues and plasma were stored at −80 °C for later analysis.

### Western blot analysis

Antibodies against IRS-2 (CST #4502, 1/1000, Boston, USA), IRS-1 (CST #2390, 1/1000, Boston, USA), p-IRS-2 (Abcam #ab3690, 1 µg/ml, London, England), p-IRS-1 (CST #3203, 1/1000, Boston, USA), Akt (CST #4685, 1/1000, Boston, USA), p-Akt (CST #4058, 1/1000, Boston, USA), Glut 2 (Abcam #ab54460, 5 µg/ml, London, England), Glut 4 (Abcam #ab654, 1/2000, London, England), IκBα (CST #4814, 1/1000, Boston, USA), p-IκBα (CST #9246, Boston, 1/1000, USA), p-NF-κB p65 (CST #3033, 1/1000, Boston, USA) were prepared, and separated proteins (30 μg total) from liver, skeletal muscle and adipose tissue reacting with these monoclonal or polyclonal antibodies were detected by western blot. β-Actin (CST #12620, 1/1000, Boston, USA) or GAPDH (Abcam #ab8245, 1/5000, London, England) was used as a total protein loading control, Na-K ATPase (Abcam #ab185210, 1/5000, London, England) was used as the membrane protein loading control, and Histone H3 (Abcam #ab8580, 1 µg/ml, London, England) was used as the nucleoprotein loading control. Liver, skeletal muscle and adipose tissues were lysed with RIPA lysis buffer (Boster Biological Technology Co., Ltd., Wuhan, China) and then centrifuged at 14,000 rpm and 4 °C for 15 min. A membrane protein extraction kit (Boster Biological Technology CO., Ltd, Wuhan, China) was used to extract membrane proteins, and a nucleoprotein kit (Boster Biological Technology Co., Ltd., Wuhan, China) was used to extract nuclear proteins. The protein concentrations were quantified by a BCA protein assay kit (Boster Biological Technology Co., Ltd., Wuhan, China). Equal amounts of protein (30 µg) were separated by 10% SDS/PAGE and then transferred to a PVDF membrane. After blocking with 5% nonfat dry milk, the membranes were incubated with primary antibody overnight at 4 °C, washed with TBST and incubated with horseradish peroxidase (HRP)-conjugated secondary antibody for an additional 2 h at room temperature. The blots were visualized by an enhanced chemiluminescence western blot detection system (Beyotime Biotechnology, Shanghai, China). The signal intensity was quantified using ImageJ software.

### Immunofluorescence staining

After dewaxing, the slices were washed 3 times in PBS, permeabilized in 0.1% Triton for 10 min, subjected to antigen retrieval in citric acid solution, blocked with 5% BSA for 1 h and incubated with a primary antibody (Glut-4, Abcam, ab654, 1/1000; p-NF-κB p65, CST #3033, 1/100) overnight at 4 °C. The next day, the secondary antibody (FITC-conjugated goat anti-rabbit IgG, ZSGB-BIO, ZF-0311, 1/150) was added to the sections. The nucleus was stained by DAPI. The slices were imaged using a fluorescence microscope (Olympus Corporation, Japan).

### Real-time PCR

The *TNF-α* and *IL-6* mRNA levels in liver, skeletal muscle and adipose tissue were determined by real-time PCR. Snap-frozen rat liver, skeletal muscle and epididymal fat tissues were homogenized in Trizol (Ambion RNA, Carlsbad, CA), and the RNA was extracted. cDNA was generated from each RNA sample by reverse transcription with the M-MLV RTase cDNA kit according to the manufacturer’s instruction. Real-time PCR was performed using the SYBR Premix EX Tap 2x kit in a CFX 96 PT-PCR system. The reaction conditions were as follows: 95 °C for 30 s, followed by 40 cycles of 95 °C for 5 s and 60 °C for 30 s, and ending with 72 °C for 10 min. Gene expression was normalized to the *β-actin* and *GAPDH* housekeeping genes. PCR primers were designed based on previous publications or using online primer design software (Primer 3 Input; http://primer3.ut.ee/) combined with NCBI and gene editing software DNAMAN. Primer sequences were as follows:

*TNF-*α^45^: F: 5′-TACTGAACTTCGGGGTGATTGGTCC-3′;

R: 5′-CAGCCTTGTCCCTTGAAGAGAACC-3′;

*IL-6*^46^: F: 5′-TCCTACCCCAACTTCCAATGCTC-3′;

R: 5′-TTGGATGGTCTTGGTCCTTAGCC-3′;

*β-actin*: F: 5′-CGTTGACATCCGTAAAGACC-3′;

R: 5′-AGAGCCACCAATCCACACA-3′;

*GAPDH*: F: 5′-GTGGAGTCTACTGGCGTCTT-3′;

R: 5′-CCTGCTTCACCACCTTCTTG-3′.

### Statistical analysis

Data analysis was performed with SPSS 20.0 (SPSS Inc., Chicago, IL, USA). The results are presented as the mean ± SD. If the data were normally distributed with a homogeneous variance, differences between two groups were assessed with unpaired and two-tailed Student’s t tests. One-way analysis of variance followed by the least significant difference (LSD) t-test and Tukey’s test was used to calculate differences among various study groups. If the data did not obey a normal distribution or the variance was not uniform, a non-parametric test (Kruskal-Wallis test) was used to analyse differences between groups. *P* < 0.05 indicated statistical significance.

## Electronic supplementary material

Supplementary Information

## Data Availability

The results of calculations can be obtained by request from the authors.
