# Supplementary material for: Effects of Vaspin on Insulin Resistance in Rats and Underlying Mechanisms
Source: Sci Rep. 2018 Sep 10;8:13542. doi: 10.1038/s41598-018-31923-3 (PMC6131547; doi:10.1038/s41598-018-31923-3)
Supplement: Supplementary file 1 — Supplementary Information [file 41598_2018_31923_MOESM1_ESM.pdf]

Supplementary materials

Effects of Vaspin on Insulin Resistance in Rats and Underlying Mechanisms

Shiwei Liu[1,2]*, Ruixue Duan[3], Yaru Wu[3], Fang Du[3], Jiaxin Zhang[3], Xin Li[4], Shenghui Guo[3], Meimei Wang[3], Qi Zhang[3], Yuanbin Li[4], and Naishi Li[5]

1 Department of Endocrinology, Shanxi DAYI Hospital, Shanxi Medical University, Taiyuan, China.

2 Department of Central Laboratory, Taiyuan Central Hospital, Shanxi Medical University, Taiyuan, China.

3 Department of Graduate School of Shanxi Medical University, Taiyuan, China.

4 Department of Endocrinology, Taiyuan Central Hospital, Shanxi Medical University, Taiyuan, China.

5 Department of Endocrinology, Key Laboratory of Endocrinology, Peking Union Medical College Hospital, Chinese Academy of Medical Science, Beijing, China.

*Corresponding author: Shiwei Liu (lswspring6@aliyun.com)

Department of Endocrinology, Shanxi DAYI Hospital, Shanxi Medical University, Taiyuan, China (030032); Central Laboratory, Taiyuan Central Hospital, Shanxi Medical University, Taiyuan, China (030009)

Supplemented information

p-IRS-2

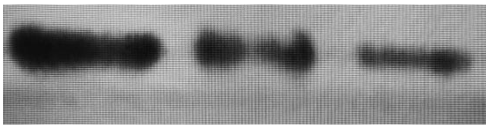

IRS-2

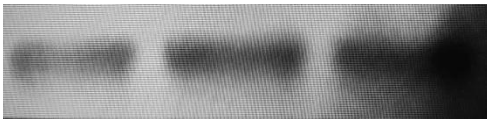

p-Akt

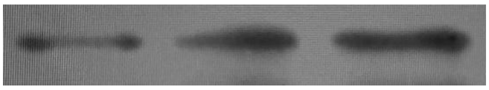

Akt

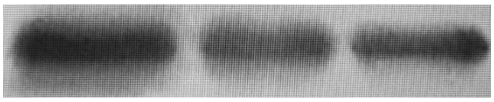

β-actin

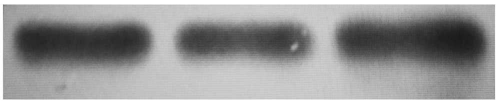

Figure S1. Original scans for the western blots in Figure 4A.

Membrane Glut-2

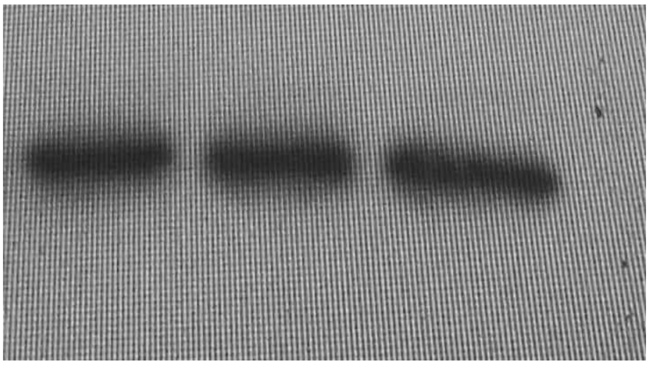

Na-K ATPase

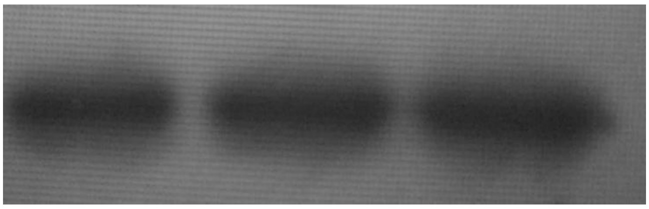

Cytoplasmic Glut-2

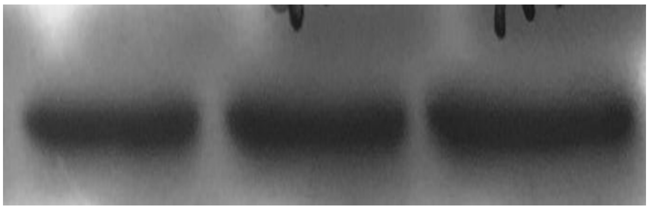

β-actin

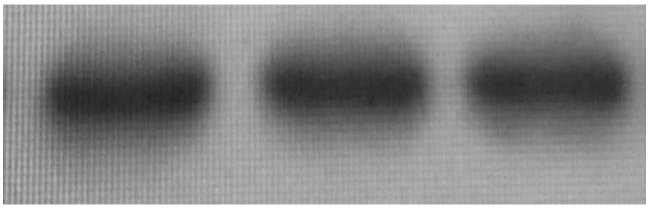

Figure S2. Original scans for the western blots in Figure 4E.

p-IRS-1

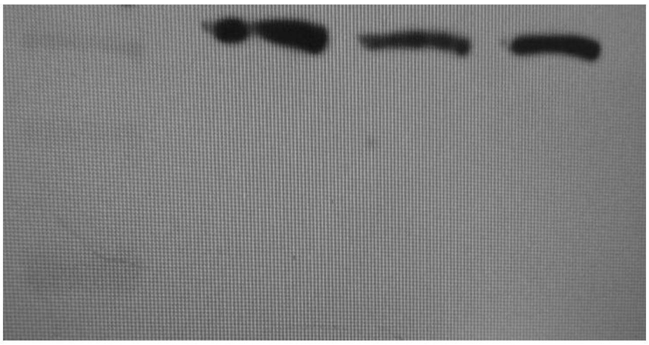

IRS-1

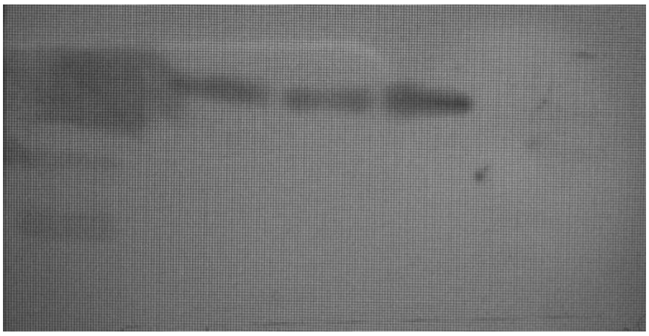

p-Akt

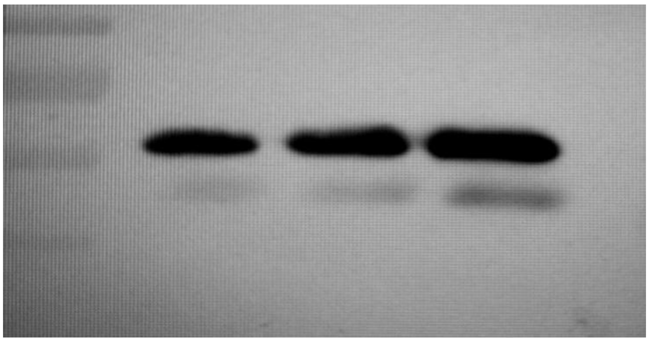

Akt

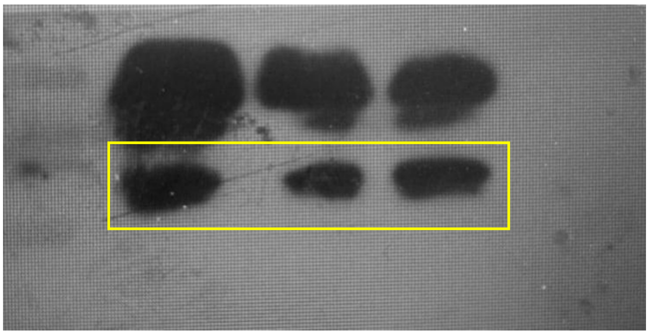

GAPDH

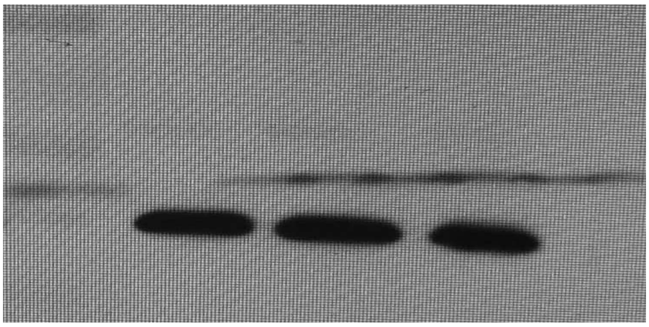

Figure S3. Original scans for the western blots in Figure 5A.

Membrane Glut-4

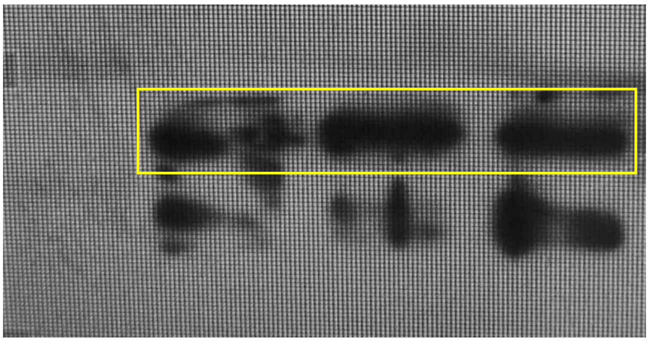

Na-K ATPase

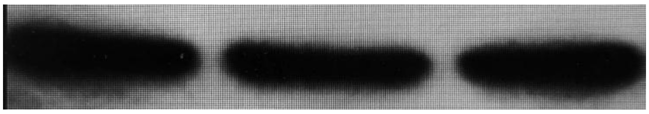

Cytoplasmic Glut-4

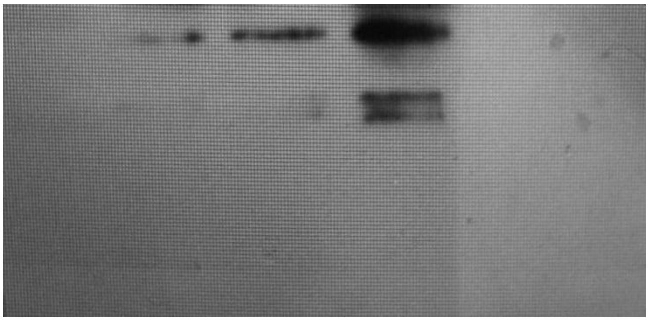

GAPDH

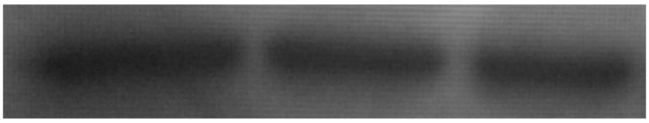

Figure S4. Original scans for the western blots in Figure 5E.

p-IRS-1

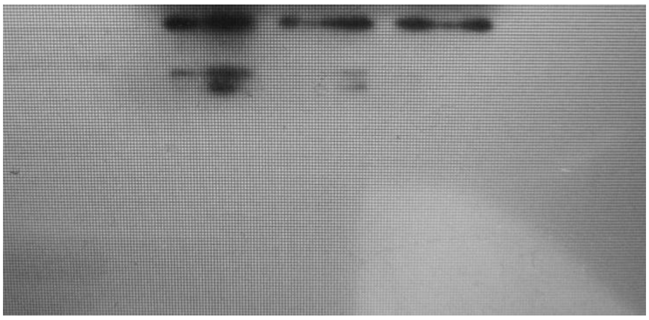

IRS-1

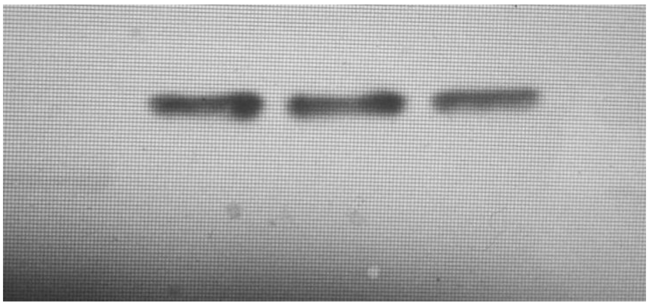

p-Akt

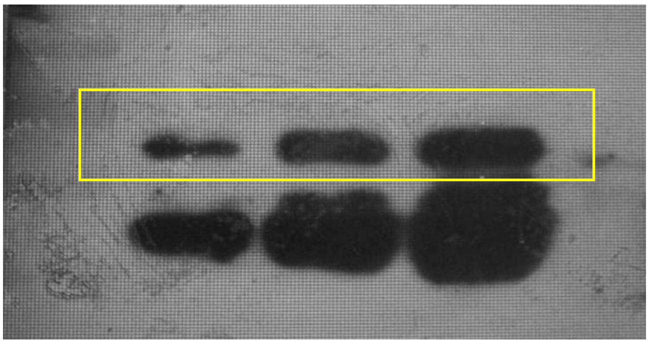

Akt

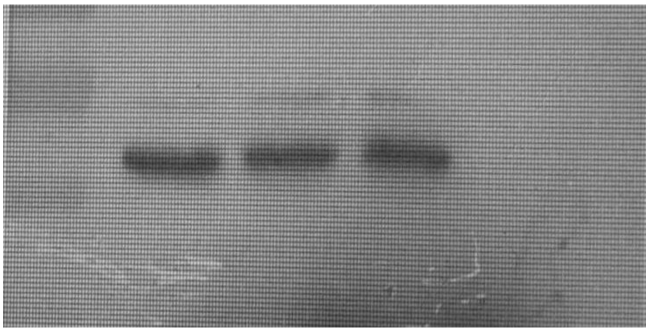

β-actin

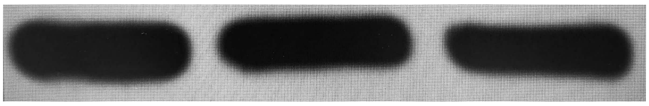

Figure S5. Original scans for the western blots in Figure 6A.

Membrane Glut-4

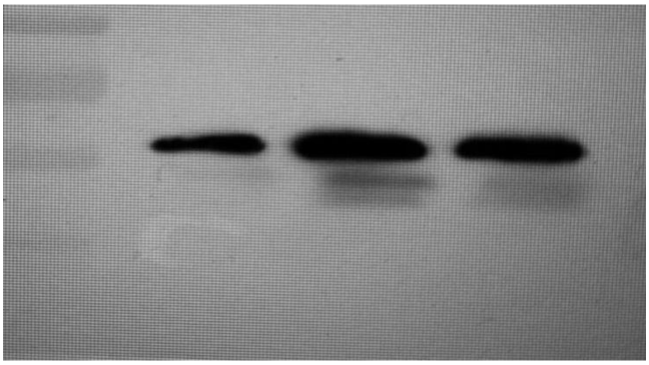

Na-K ATPase

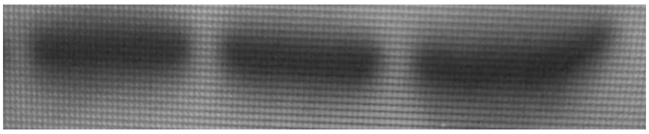

Cytoplasmic Glut-4

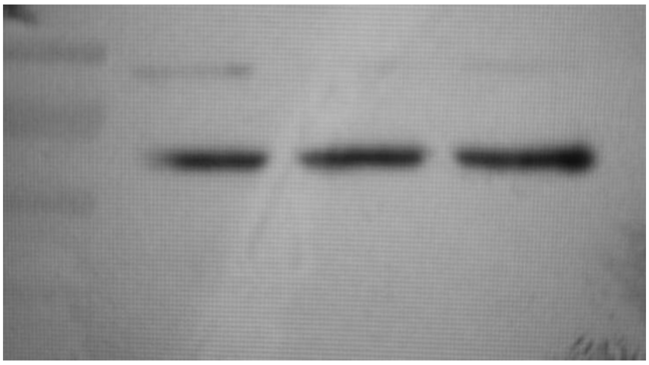

GAPDH

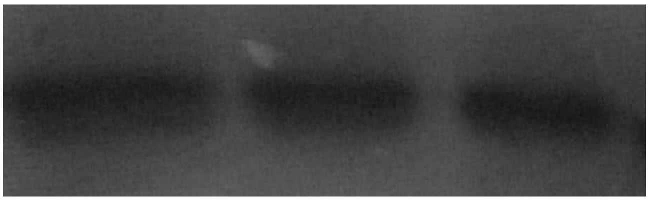

Figure S6. Original scans for the western blots in Figure 6E.

p-IκBα

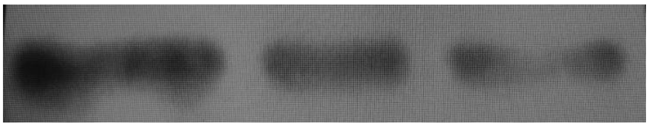

IκBα

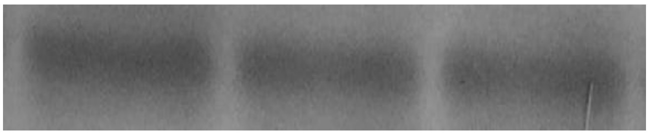

β-actin

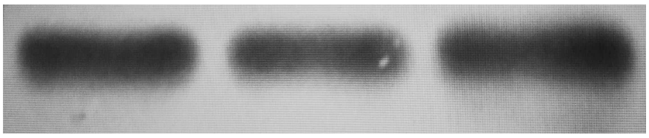

Figure S7. Original scans for the western blots in Figure 7A.

p-NK-κB p65

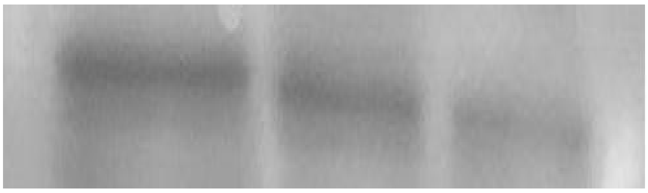

Histone H3

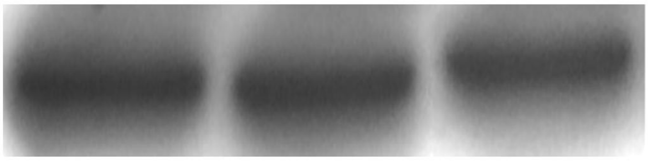

Figure S7. Original scans for the western blots in Figure 7C.

p-IκBα

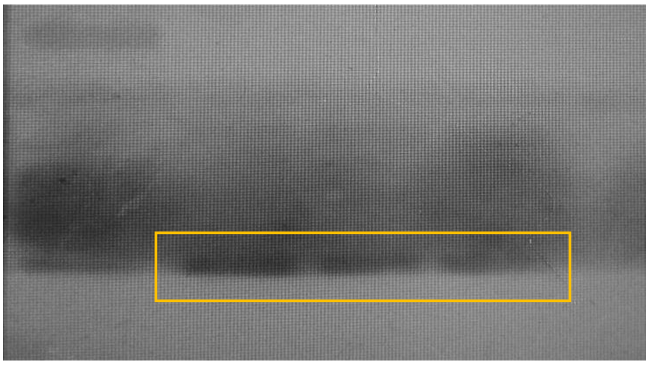

IκBα

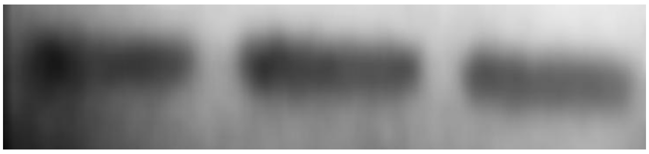

GAPDH

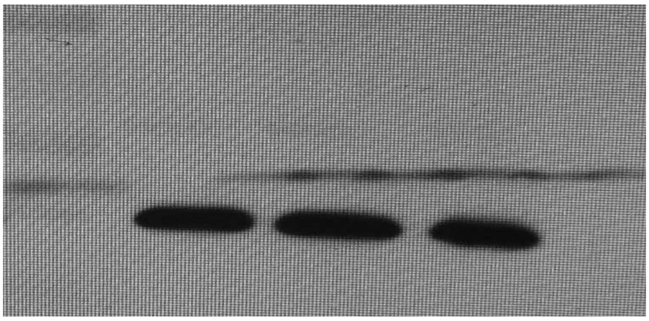

Figure S8. Original scans for the western blots in Figure 8A.

p-NK-κB p65

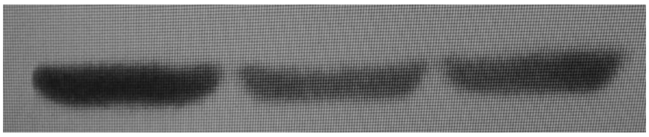

Histone H3

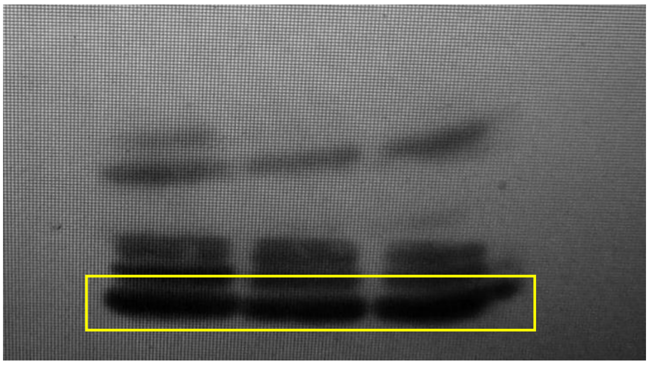

Figure S9. Original scans for the western blots in Figure 8C.

p-IκBα

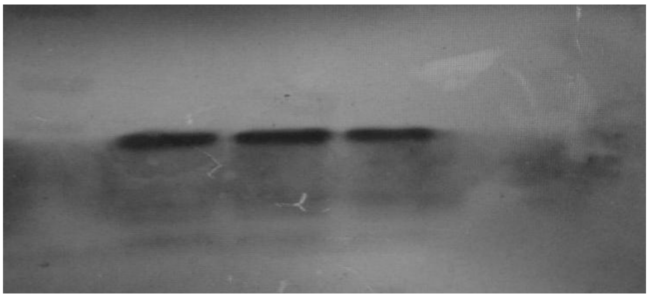

IκBα

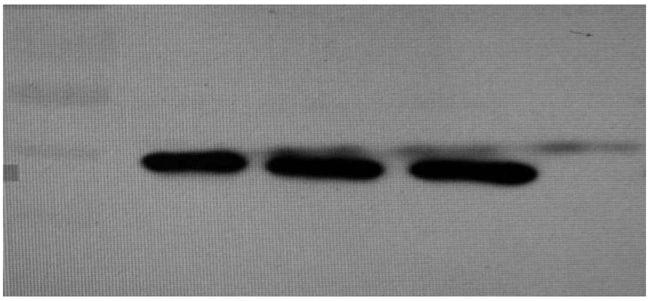

β-actin

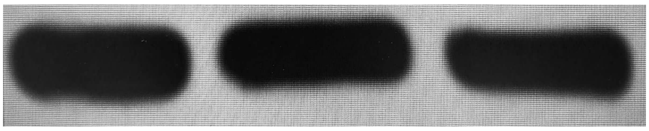

Figure S10. Original scans for the western blots in Figure 9A.

p-NK-κB p65

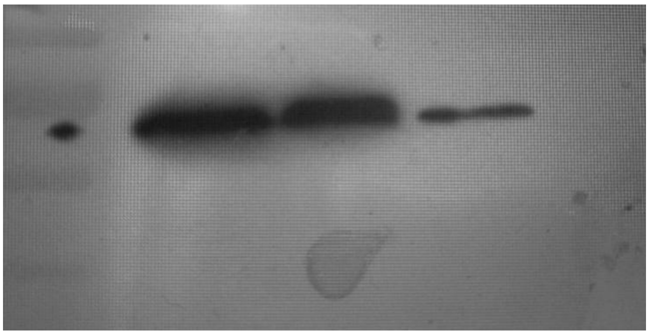

Histone H3

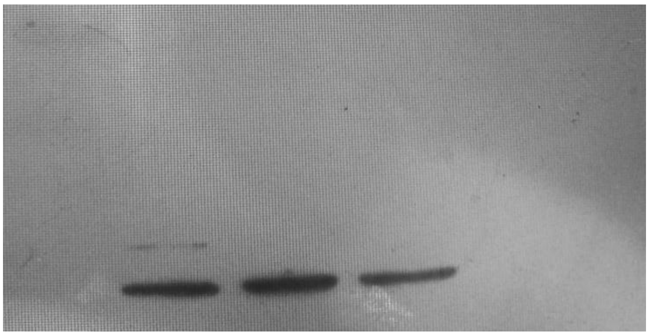

Figure S11. Original scans for the western blots in Figure 9C.

	HFD	HFD+vaspin	NC
Weight (g)	457±31*	440±29	386±24
FBG (mmol/L)	6.43±0.72*	5.81±0.60#	5.17±0.49
FINS (mIU/L)	64.8±13.1*	47.9±10.3#	37.5±8.6
TG (mmol/L)	0.83±0.09*	0.82±0.10	0.52±0.05
TC (mmol/L)	1.39±0.11*	1.41±0.11	1.19±0.06
TNF-α (pg/ml)	109.64± 11.73*	78.55± 10.67#	29.43 ± 5.18
IL-6 (pg/ml)	157.34±23.17*	102.58±20.45#	85.12±14.26

**P* < 0.05 compared with the NC group; #*P* < 0.05 compared with the HFD group.

Table S1. Numerical values of Fig 1A-G.

	HFD	HFD+vaspin	NC
AUC of IPGTT	16.265±1.055*	14.385±0.742#	12.950±0.929
AUC of ITT	146.83±12.73*	130.32±6.38#	117.95±12.39
GIR(mg/kg·min)	9.132±1.673*	13.932±1.717#	19.378±1.982

**P* < 0.05 compared with the NC group; #*P* < 0.05 compared with the HFD group.

Table S2. Numerical values of Fig 2B, 2E and 3C.

	HFD	HFD+vaspin	NC
p-IRS-2	0.84±0.09*	0.61±0.07#	0.49±0.07
IRS-2	0.38±0.06*	0.57±0.08#	0.56±0.08
p-Akt	0.32±0.05*	0.58±0.07#	0.70±0.07
Akt	1.32±0.10*	1.12±0.10#	1.10±0.10
p-IRS-2/IRS-2	2.211±0.125*	1.070±0.097#	0.804±0.076

p-Akt/Akt	0.242±0.035*	0.518±0.057#	0.636±0.078
Membrane Glut-2	0.524±0.076*	0.623±0.079	0.998±0.101
Cytoplasmic Glut-2	0.53±0.06*	0.72±0.07	1.02±0.09

**P* < 0.05 compared with the NC group; #*P* < 0.05 compared with the HFD group.

Table S3 Numerical values of Fig 4B, 4C, 4D, 4F.

	HFD	HFD+vaspin	NC
p-IRS-1	0.785±0.098*	0.373±0.075#	0.558±0.099
IRS-1	0.315±0.064*	0.223±0.059	0.728±0.067
p-Akt	1.419±0.196*	1.794±0.231	2.284±0.285
Akt	1.593±0.137*	0.813±0.096#	1.289±0.157
p-IRS-1/IRS-1	2.493±0.256*	1.672±0.167#	0.767±0.096
p-Akt/Akt	0.840±0.089*	2.202±0.157#	1.768±0.136
Membrane Glut-4	0.337±0.046*	0.775±0.068#	0.925±0.085
Cytoplasmic Glut-4	0.051±0.042*	0.195±0.033#	0.894±0.085

**P* < 0.05 compared with the NC group; #*P* < 0.05 compared with the HFD group.

Table S4 Numerical values for Fig 5B, 5C, 5D, 5F.

	HFD	HFD+vaspin	NC
p-IRS-1	0.468±0.034*	0.157±0.050#	0.153±0.034
IRS-1	0.362±0.036	0.321±0.038	0.291±0.074
p-Akt	0.149±0.035*	0.403±0.046#	0.629±0.047
Akt	0.213±0.042	0.210±0.039	0.237±0.035
p-IRS-1/IRS-1	1.293±0.087*	0.490±0.069#	0.525±0.053
p-Akt/Akt	0.699±0.091*	1.921±0.148#	2.657±0.187
Membrane Glut-4	0.267±0.065*	0.620±0.073#	0.543±0.079

Cytoplasmic Glut-4	0.367±0.043*	0.600±0.067	0.922±0.082

*$P < 0.05$ compared with the NC group; #$P < 0.05$ compared with the HFD group.

Table S5 Numerical values of Fig 6B, 6C, 6D, 6F.

	HFD	HFD+vaspin	NC
p-IκBα/ IκBα	0.658±0.079*	0.448±0.059#	0.246±0.043
p-NF-κB p65	0.702±0.101*	0.491±0.090#	0.253±0.071
TNF mRNA	3.072±0.484*	2.202±0.219#	1.374±0.160
IL-6 mRNA	2.302±0.361*	1.970±0.038#	1.326±0.178

*$P < 0.05$ compared with the NC group; #$P < 0.05$ compared with the HFD group.

Table S6 Numerical values of Fig 7B, 7D, 7E, 7F.

	HFD	HFD+vaspin	NC
p-IκBα/ IκBα	0.476±0.036*	0.269±0.029#	0.198±0.028
p-NF-κB p65	0.661±0.071*	0.451±0.082#	0.483±0.029
TNF mRNA	2.692±0.432*	2.002±0.399#	1.374±0.160
IL-6 mRNA	2.330±0.429*	1.570±0.357#	1.326±0.178

*$P < 0.05$ compared with the NC group; #$P < 0.05$ compared with the HFD group.

Table S7 Numerical values of Fig 8B, 8D, 8F, 8G.

	HFD	HFD+vaspin	NC
p-IκBα/ IκBα	0.920±0.086*	0.662±0.079#	0.326±0.062
p-NF-κB p65	4.020±0.245*	2.670±0.160#	1.010±0.097
TNF mRNA	1.476±0.246*	1.060±0.079#	0.534±0.164
IL-6 mRNA	1.998±0.158*	1.450±0.248#	1.326±0.178

*P < 0.05 compared with the NC group; #P < 0.05 compared with the HFD group.

Table S8 Numerical values of Fig 9B, 9D, 9F, 9G.
